# Animal and cellular models of atrial fibrillation: a review

**DOI:** 10.3389/fcvm.2025.1617652

**Published:** 2025-08-11

**Authors:** Qiuying Wu, Xize Wu, Teng Feng, Feiyu Chen, Jiaqi Ren, Shan Gao, Bo Wang, Yue Li, Lihong Gong

**Affiliations:** ^1^ The First Clinical College, Liaoning University of Traditional Chinese Medicine, Shenyang, Liaoning, China; ^2^ Department of Cardiology, Affiliated Hospital of Liaoning University of Traditional Chinese Medicine, Shenyang, Liaoning, China

**Keywords:** atrial fibrillation, animal model, cellular model, biomarkers, review

## Abstract

Modeling atrial fibrillation (AF) is crucial for investigating its pathogenesis and developing new therapeutic strategies. To better explore the mechanisms underlying AF and promote the progress of basic research, it is particularly important to develop accurate animal models that closely simulate the progression of clinical disease. This review summarizes the methods and evaluation criteria for establishing animal and cellular AF models over the past decade, highlighting the advantages and limitations of various models to provide a reference for basic research and treatment of AF. Current experimental animals are primarily categorized into small animals (mice, rats, rabbits), large animals (dogs, pigs, sheep, horses), and model organisms (zebrafish), with modeling methods including electrophysiological induction, chemical induction, trauma induction, and genetic editing. Cellular models commonly use primary cultured cardiomyocytes, the HL-1 cell line, hiPSC-CMs, and H9c2 cells as subjects of study. However, due to the lack of standardized modeling protocols, researchers evaluate AF models based on electrophysiological properties, atrial functional metrics, and biomarkers. Three-dimensional engineered tissues and artificial intelligence, as emerging fields, play an important role in the diagnosis, treatment, and prognostic monitoring of AF. This paper not only summarizes the current progress in AF model research but also points out the deficiencies of existing models, offering guidance for future research directions.

## 1 Introduction

Atrial fibrillation (AF) is one of the most prevalent cardiac arrhythmias, associated with significant disability and mortality. Global epidemiological studies have indicated that there are over 33 million AF patients worldwide, with a notable increase in prevalence with advancing age (1, 2). Moreover, individuals with AF face a 1.5 to 1.9 times higher risk of mortality compared to those without AF (3). This elevated risk is intrinsically linked to the frequent coexistence of AF with underlying cardiovascular pathologies and risk factors. Key contributors include hypertension, heart failure, valvular heart disease, coronary artery disease, diabetes, obesity, and the development of atrial cardiomyopathy. Atrial cardiomyopathy refers to the progressive structural, electrical, and functional remodeling of the atrial myocardium (characterized by fibrosis, inflammation, ion channel dysfunction, and contractile impairment), which not only serves as a critical substrate facilitating AF initiation and perpetuation but also independently contributes to adverse outcomes like stroke and heart failure (4). Despite the existence of several theoretical hypotheses aiming to explain the pathogenesis of AF, including focal activation, multiple wavelet reentry, and rotor theory (5–7), these theories have yet to fully unravel the intricate mechanisms driving AF development. Therefore, a thorough investigation into the molecular mechanisms and pathophysiological processes of AF and exploration of novel therapeutic strategies is imperative for reducing AF incidence, enhancing patient outcomes, and improving their quality of life, which holds substantial clinical and societal importance.

The establishment of disease models is a key link in modern biomedical research and plays an indispensable role in the observation and investigation of disease phenomena. By simulating clinical disease prototypes, the establishment of stable animal or cell models with symptoms and pathological changes similar to clinical diseases is essential for the treatment and further research of complex clinical diseases. Research has shown that large animals, due to their cardiac anatomy and electrophysiological properties akin to humans, exhibit a higher propensity for spontaneous AF (8). However, the application of large animals to establish AF models mostly requires invasive procedures such as open-chest pacemaker placement, which presents challenges including high operational requirements, substantial experimental costs, and significant inter-individual differences, potentially undermining the reliability of research findings. In contrast, small animal models offer distinct advantages, including well-defined genomic information that facilitates gene editing and molecular research, lower experimental costs, highly standardized operational procedures, and shorter reproductive cycles, which facilitates large-scale studies and makes small animal models invaluable for investigating the genetic and molecular pathological mechanisms of AF (9). Furthermore, *in vitro* cell cultures have emerged as a crucial tool for exploring the molecular mechanisms of AF, thanks to their operational simplicity, reproducibility, and standardized assay methods (10).

Therefore, this paper provides a comprehensive review of the past decade's advancements in the selection of animals for AF modeling, the methodologies for establishing animal and cellular models of AF, and the criteria for evaluating these models. The aim is to offer a reference that could aid in both the scientific inquiry and clinical management of AF.

## 2 Selection of experimental animals for AF models

Investigating the pathogenesis and developing therapeutic strategies for AF heavily rely on the use of animal models. Given the diversity of available AF model animals, selecting the appropriate species based on experimental requirements and practical considerations is crucial for conducting meaningful research. AF models are primarily categorized into small animal (e.g., rodents, rabbits), large animal (e.g., dogs, pigs, sheep, horses), and model organism (e.g., zebrafish) systems, each offering distinct advantages and limitations (Table 1).

**Table 1:** Advantages and disadvantages of experimental animals in animal models of AF.

Animal	Animal species	Advantage	Disadvantage
Small animal	Mouse	Easy breeding, lower cost, abundant strains, clear genetic background, and amenability to genetic modification.	Smaller heart size, more difficult maneuverability, and greater differences in heart structure from humans make it impossible to mimic the electrophysiologic heterogeneity of human pulmonary veins, difficult to develop spontaneous AF requires electrical stimulation, chemical induction, or gene editing.
	Rat	Easy breeding, lower cost, larger heart size than mice, relatively easy experimental manipulation, closer to humans than mice in cardiovascular, neurological, and drug metabolism.	Lack of pulmonary venous muscular sleeve structures, minimal spontaneous atrial fibrillation that is largely induced, and a heart too small for interventional procedures such as catheter ablation limit its translational research value.
	Rabbit	Distinct action potential characteristics, consistent electrophysiological changes with humans, small body size, easy breeding, and relatively simple experimental procedures.	Short duration of AF, few genetic modifications.
Large animal	Dog	Presence of spontaneous AF, electrophysiologic characteristics similar to humans, ease of use of clinical instruments.	Higher breeding costs, requires specialized breeding and management conditions, low social acceptance.
	Pig	The heart and coronary artery structure are similar to humans, and there are numerous enzymes and proteins that are identical to those in humans, ease of use of clinical instruments.	Higher breeding costs, requires specialized breeding and management conditions, time-consuming experiments.
	Sheep and goats	Better tolerance to AF, ease of use of clinical instruments.	Higher breeding costs, requires specialized breeding and management conditions, time-consuming experiments, complex experimental procedures, large individual differences.
	Horse	Presence of spontaneous AF, ease of use of clinical instruments.	Higher breeding costs, requires specialized breeding and management conditions, low social acceptance, time-consuming experiments, complex experimental procedures, large individual differences, electrophysiological characteristics different from humans, different action potentials with different durations.
Model organism	Zebrafish	Strong reproductive capacity, rapid development, low breeding costs, large spawning capacity facilitating experimental material collection, *in vitro* fertilization, *in vitro* development, transparency of the fish body in early development stages facilitating experimental manipulation, and high similarity to humans in terms of the nervous system.	Significant differences in anatomy and physiological function with humans.

### 2.1 Small animal AF models

Rodents (mice/rats) and rabbits are the most widely utilized small animals in AF research. While rodents rarely develop spontaneous AF and typically require external induction methods (e.g., pacing, pharmacological stimulation), they are highly utilized in experimental studies due to their lower maintenance costs, high experimental efficiency, and broad social acceptance (11, 12). Mice are particularly favored for genetic and molecular studies because of their well-characterized genomes, high gene homology with humans, and the availability of diverse transgenic strains. Rats are preferred for complex surgical and behavioral experiments given their larger size and closer anatomical resemblance to humans. Moreover, the pulmonary vein diameter in rodents is only about 0.1–0.5 mm, which is significantly smaller compared to that in humans (10–20 mm), making it impossible for catheter intervention. Additionally, the pulmonary veins in humans are surrounded by a “muscular cuff” extended from the atrial myocardium, which is a key structure for AF triggers. However, the muscular cuff of the pulmonary veins in rodents is extremely short or even completely absent, and thus they cannot simulate the electrophysiological heterogeneity of human pulmonary veins (13–15). Rabbits offer significant advantages in cellular electrophysiology, with action potential characteristics closely resembling those of humans, especially in studies related to ion channel function, repolarization and re-entrant ventricular tachycardia (16). This similarity makes rabbits particularly valuable for studies related to the electrophysiological mechanisms of AF.

### 2.2 Large animals AF models

Large animals more closely replicate human AF pathophysiology due to their cardiac size and electrophysiological complexity. Dogs are frequently used in rapid pacing models, with older and male dogs exhibiting a higher propensity for spontaneous AF. Their larger heart size and faster heart rates make them suitable for studying AF's electrophysiological and pathophysiological processes (8, 17). Pigs, despite no reports of spontaneous AF, are excellent for modeling human diseases due to their anatomical and physiological similarities, particularly miniature pigs, which closely resemble human organs in size, structure, and function (8). Goat models, while costly and complex, benefit from the high tolerance of sheep to sustained AF, enabling the study of chronic AF mechanisms (18). In sheep, the presence of an aortopulmonary shunt makes their atria more susceptible to electrophysiological changes and structural remodeling, thereby increasing their susceptibility to AF (19). Horses, which often experience persistent AF and have a reported spontaneous AF incidence of about 2.5%, offer a unique natural model for investigating long-term AF management strategies (20, 21).

### 2.3 Model organism AF models

Zebrafish, as a novel vertebrate model organism intermediate between cells and mammals, serve as an ideal model for AF research. The embryonic transparency of zebrafish allows researchers to directly perform gene editing injections at the single-cell stage. Coupled with advanced optical voltage mapping and calcium transient dynamics analysis techniques, this enables real-time visual observation of cardiac electrical activity (22). The reproductive biology of zebrafish, characterized by external fertilization and external development, facilitates the ability to manipulate key reproductive traits (e.g., precisely controlling fertilization timing and screening embryos), offering significant experimental advantages. These features not only circumvent the ethical constraints associated with mammalian *in utero* manipulation but also allow for the generation of hundreds of gene-edited individuals in a single experiment. This is attributed to the large spawn size and convenient access to experimental materials, greatly enhancing research throughput and efficiency.

Additionally, the zebrafish heart is highly conserved in electrophysiological properties with respect to human (23), the action potentials of the zebrafish ventricle and those of the human heart both exhibit a long plateau phase, resulting in a distinct QT interval in the zebrafish ECG. Furthermore, significant similarities in the major ionic current systems, such as sodium current (INa), L-type calcium current (ICaL), and potassium current (IK), are observed between the two. These similarities position zebrafish as a valuable model for studying human cardiac electrophysiology (24). However, despite these notable similarities in electrophysiological characteristics, there are key differences between zebrafish and human hearts that may impact the applicability of zebrafish as a model for human cardiac electrophysiological mechanisms. Firstly, zebrafish cardiac electrophysiological mechanisms involve activation from the apex to the base and depolarization from the base to the apex, whereas human cardiac electrophysiological mechanisms exhibit the opposite pattern, with activation from the base to the apex and depolarization from the apex to the base (25). Secondly, zebrafish ventricles possess a large T-type calcium current (ICaT), which is absent in human ventricles (26). Furthermore, the composition of potassium currents also differs: the inwardly rectifying potassium current (IK1) in zebrafish is primarily mediated by Kir2.4 and Kir2.2a channels, while in humans, IK1 is predominantly generated by Kir2.1 and Kir2.4 channels (27). These key differences indicate that while zebrafish can serve as a powerful model for studying human cardiac electrophysiology in certain aspects, there are limitations in replicating specific mechanisms of the human heart. Therefore, when utilizing the zebrafish model for relevant studies, it is essential to consider these differences to ensure the accuracy and applicability of the results.

In summary, the selection of model animals should be based on experimental objectives, environmental conditions, funding, and the strengths and weaknesses of each model. Small animals (e.g., rodents, rabbits) offer cost efficiency, genetic tractability, and high-throughput screening potential, making them ideal for mechanistic and early-stage drug studies. Large animals (e.g., dogs, pigs, sheep) better replicate human cardiac physiology and are essential for translational research, including ablation techniques and chronic AF modeling. Zebrafish, with their optical transparency and high gene-editing efficiency, serve as a powerful tool for rapid genetic screening and developmental electrophysiology. A tiered approach—using small animals or zebrafish for target discovery and large animals for preclinical validation—optimizes both efficiency and clinical relevance in AF research. Future studies should integrate multiple models to bridge molecular insights with therapeutic applications.

## 3 Construction of AF animal models

AF remains a critical focus in both clinical and scientific communities due to its complex pathogenesis, high incidence, notable recurrence rates, and associated mortality risks from complications (28). The development of accurate AF models is essential for advancing experimental research. Various modeling techniques have been developed and refined, including electrophysiological induction, chemical induction, and trauma induction. However, variability in drug protocols and electrophysiological parameters can lead to inconsistent success rates in establishing these models. Therefore, the careful selection of appropriate modeling techniques is crucial for ensuring the validity and reliability of research outcomes.

### 3.1 Electrophysiological induction

Electrophysiological induction is recognized as the most extensively utilized and versatile approach for establishing AF models across various species. Based on the anatomical location and approach, this technique can be categorized into direct atrial stimulation and transesophageal electrical stimulation.

#### 3.1.1 Direct atrial stimulation

Direct atrial stimulation typically involves percutaneous insertion of a multipolar catheter through the internal jugular vein to deliver controlled electrical stimulation to the right atrium. By simulating the electrophysiological changes characteristic of clinical AF, this approach effectively induces both electrical and structural remodeling in atrial myocardium. The key pathophysiological consequences include significant shortening of the atrial effective refractory period (AERP) and increased atrial susceptibility to arrhythmogenesis, thereby reliably triggering AF episodes (29).

The underlying mechanisms involve complex alterations in cardiomyocyte electrophysiology. Electrical stimulation induces massive opening of voltage-gated sodium and calcium channels, leading to abnormal intracellular ion concentrations. This rapid increase in intracellular sodium and calcium ion concentrations causes an abnormal depolarization process in cardiomyocytes. Additionally, electrical stimulation affects the function of potassium ion channels, disrupting the repolarization process and leading to changes in the action potential time course and the refractory period of cardiomyocytes. These alterations in electrophysiological properties make the excitability and conductivity of cardiomyocytes abnormal, creating conditions conducive to the development of AF.

Three primary electrophysiological techniques are widely used in AF research: programmed electrical stimulation, burst pacing, and rapid atrial pacing. Programmed electrical stimulation utilizes a basic stimulus (S1) followed by progressively premature extrastimuli (S2) to systematically decrease the S1S2 coupling interval until AF induction. The basic stimulus-delivered pacing modes include (1) burst pacing with fixed cycle lengths, (2) decremental pacing, where the pacing cycle length is gradually shortened during stimulation, and (3) the introduction of premature beats or additional stimuli during sinus rhythm or ongoing pacing. Burst pacing employs short bursts of high-frequency stimulation to rapidly induce acute AF episodes. In contrast, rapid atrial pacing involves continuous high-frequency pacing over extended periods, making it particularly valuable for studying chronic AF and associated remodeling processes.

Despite its widespread use, significant variability exists in stimulation protocols across studies. Critical parameters, including pacing pattern, stimulation amplitude, pulse duration, and overall pacing duration, remain unstandardized (30). This methodological heterogeneity poses challenges for result comparison and reproducibility, highlighting the need for standardized protocols in AF model establishment. The selection of specific stimulation parameters should be carefully considered based on the particular research objectives, whether focused on acute arrhythmia induction or chronic remodeling processes (Table 2).

**Table 2:** Af model induced by direct atrial stimulation.

Author	Preprocessing	Electrophysiologic pacing parameters	Stimulating area	Animal species	Definition of AF	Induction success rate	Purpose of the study
Chen et al. (31)	Angiotensin II was injected by a subcutaneous osmotic pump at 1,800 ng/kg/min (2.592 mg/kg/d) for 28 d.	The burst pacing parameters were set to an amplitude of 3 V, a pulse width of 2 ms, and a total of 10 pacing episodes.	Right jugular vein.	C57BL/6 mice	≥2 s; Irregular	9/10; 90%	Macrophage infiltration and inflammation.
Jansen et al. (32)	Angiotensin II was injected by a subcutaneous osmotic pump at 3 mg/kg/d for 21 d.	The burst pacing parameters were set to a current of 0.4 mA and a pulse width of 2 ms.	Catheter was inserted into the right heart.	C57BL/6 mice	≥1 s; Irregular	12/24; 50%	Atrial electrical and structural remodeling.
Chen et al. (33)	Angiotensin II was injected by a subcutaneous osmotic pump at 0.11 μl/h (2.64 mg/kg/d) for 21 d.	Intracardiac pacing (specific parameters unknown)	Catheter was inserted into mice from right jugular vein and pushed into right atrium and ventricle	C57BL/6 mice	≥1 s; Irregular	-	Atrial fibrosis, inflammation, and oxidative stress.
Sang et al. (34)	Angiotensin II was intraperitoneally injected at 0.15 mg/kg/d for 21 d.	The heart was rapidly paced for 5 s. The burst pacing cycle length gradually decreased by 2 ms from the initial 40 ms until 20 ms.	Catheter was inserted into the right atrium of the mice through the right external jugular vein.	C57BL/6J mice	≥1 s; Irregular	9/10; 90%	Mitophagy and mitochondrial stress.
Li et al. (35)	Angiotensin II was injected by a subcutaneous osmotic pump at 2,000 ng/kg/min (2.88 mg/kg/d) for 21 d.	The first 5 s burst had a cycle length of 40 ms, decreasing in each successive burst with a 2 ms decrement down to a coupling interval of 20 ms as described in previous studies. A series of bursts was repeated 3 times after stabilization for 5 min.	Catheter was inserted into the right heart.	Wild-Type Mice	≥0.6 s; Irregular	3/15; 20%	Calcium homeostasis and IK1 currents.
Yao et al. (36)	-	AF starting with 2 s burst pacing at a coupling interval of 40 ms and decreasing in each successive burst by a 2 ms decrement to a coupling interval of 10 ms.	-	Wild-Type Mice	≥1 s; Irregular	2/13; 15.4%	NLRP3 inflammasome signaling.
Yuan et al. (37)	Angiotensin II was intraperitoneally injected at 1 mg/kg/d for 14 d.	Programmed electrical stimulation (specific parameters unknown)	-	Sprague-Dawley Rats	≥1 s; Irregular	8/8; 100%	NLRP3 inflammasome signaling in atrial fibroblasts.
Luo et al. (38)	-	Post-opening programmed electrical stimulation (specific parameters unknown)	Catheter was placed on the right atrium.	Sprague-Dawley Rats	≥1 s; Irregular	4/13; 30.8%	Cardiac pyroptosis.
Guo et al. (39)	-	Pacing was started at 600 bpm, pulse width 2 ms, frequency 50 Hz for 8 h.	Catheter was inserted into the right atrium.	Rabbits	≥10 s; Irregular	6/10; 60%	Electrical and structural remodeling.
Li et al. (40)	-	Pacing was started at 600 bpm for 7 d.	The pacemaker electrode was attached to the right atrium.	Rabbits	≥10 s; -	-	Atrial electrical and structural remodeling.
Dong et al. (41)	-	Pacing was started at 600 bpm for 7 d.	A unipolar lead was then sutured onto the right atrium.	Rabbits	-; Irregular	6/10; 60%	Mitochondrial biogenesis and energy metabolism.
Yuan et al. (42)	-	Pacing was started at 900 bpm, pulse duration 1 ms, voltage 6 V for 8 h.	A set of electrodes were sutured to the epicardial surface of the left atrium.	Rabbits	>30 min; Irregular	5/10; Persistent AF 50%	Atrial neural remodeling.
Zhou et al. (43)	-	The dogs were paced at 450 bpm with 0.2 ms square-wave pulses at twice-threshold current for 14 d.	The distal end of the lead was positioned in the right atrium.	Dogs	-	-	Atrial remodeling and function.
Tu et al. (44)	-	Pacing was started at 400 bpm for 28 d.	-	Dogs	≥5 s; Irregular	-	Endoplasmic reticulum stress, inflammation, and apoptosis.
Zhao et al. (45)	-	Pacing was started at 400 bpm for 8 h/d for 8 weeks.	-	Dogs	>5 s; Irregular	-	Autonomic nerve activity and cholinergic anti-inflammatory pathway.
Igarashi et al. (46)	-	Pacing was started at 400 bpm.	Right atrium.	Dogs	>5 s; Irregular	-	Oxidative stress.
Leblanc et al. (47)	-	Pacing was started at 600 bpm for 7 d.	Catheter was placed across the tricuspid valve via the right femoral vein.	Dogs	-	-	Transcriptomic profiling.
Li et al. (48)	-	Pacing was started at 400 bpm for 6 weeks.	-	Dogs	-	84.16%	Adiponectin signaling in epicardial adipose tissue.
Yao et al. (36)	-	Pacing was started at 400 bpm for 7 d.	-	Dogs	-	-	NLRP3 inflammasome signaling.
Kazui et al. (49)	-	Pacing was started at 400 bpm for 6 weeks.	Atrial paced.	Pigs	-	-	Left atrial and ventricular structure and function.
Peng et al. (50)	-	A hexapolar catheter was inserted into the right jugular vein, advanced into the coronary sinus, and used as a guide for transseptal puncture and coronary sinus atrial pacing mapping.	A hexapolar catheter was inserted into the right jugular vein, advanced into the coronary sinus.	Pigs	-	-	Large animal model study.
Denham et al. (51)	-	The neurostimulator was programmed to deliver intermittent 50 Hz burst pacing in a repetitive cycle of 30 s on and 30 s off; at a minimum output of three times pacing threshold (range 3–5 V) with a pulse width of 390 ms.	Permanent pacing leads were advanced to the right ventricular apex and the right atrial appendage	Sheep	-	5/18; Persistent AF 28%	Large animal model study.
Macquaide et al. (52)	-	Pacing was started at 900 bpm over 22 weeks.	The pacing lead was inserted via the right jugular vein and fixed in the right atrial appendage.	Sheep	-	Persistent AF	Ultrastructural reorganization of ryanodine receptor clusters.
Lange et al. (53)	-	The pacing was continued until AF was sustained for more than 1 min, at which point pacing was reduced to 1 s on and 30 s off for 6 months.	A pacemaker was connected to a lead in the right atria	Goats	-	-	Large animal model study.
Linz et al. (54)	-	AF was induced and maintained by repetitive 50 Hz burst pacing of the atrium at 3× threshold every other second. All goats were paced for 6 weeks.	All animals received a right atrial endocardial pacemaker lead	Goats	-	Persistent AF	Sympathetic nervous system.
Ji et al. (55)	-	AF was induced and maintained by repetitive 50 Hz burst stimulation every other second, at four times the threshold, for 21 d.	Atrial lead implanted transjugularly and fixed in the right atrial appendage.	Goats	-	6/8; 75%	Ventricular arrhythmias.
Hesselkilde et al. (56)	-	Programmed electrical stimulation (specific parameters unknown)	Catheter placed in the right atrium through the jugular vein.	Horses	-	-	Large animal model study.
Hesselkilde et al. (57)	-	AF was initiated by burst pacing, which was continued daily (1–8 h per horse) until sustained AF was achieved. During AF, the pacemaker automatically started pacing if the atrial rate fell below 170/min, thus ensuring a minimum atrial rate of 170/min at all times.	Two leads were advanced to the heart and both were fixated in the right atrium.	Horses	>10 min; Irregular	Persistent AF	Large animal model study.

The electrophysiological induction protocols for atrial fibrillation models exhibit species-specific optimization:

① Mice predominantly utilize decremental burst pacing (40 ms→20 ms coupling interval, 2 ms decrements) at fixed amplitudes (0.4–3 V) and pulse widths (2 ms), typically delivered in 10-episode sequences with 5 min intervals. Prior to this pacing protocol, mice are pretreated with angiotensin II at a dosage of 1–3 mg/kg/day via mini-osmotic pumps for a period of 21 days to facilitate the induction of atrial fibrillation. AF success criterion: Irregular atrial rhythm ≥1 s.
② Rats lack standardized parameters in reported studies, though programmed electrical stimulation is commonly employed without detailed specifications. AF success criterion: Irregular atrial rhythm ≥1 s.
③ Rabbits rely on sustained rapid pacing (600–900 bpm) for 7 days, with higher-intensity protocols (900 bpm, 6 V) used for acute induction (≤8 h). AF success criterion: Irregular atrial rhythm ≥10 s.
④ Dogs require chronic high-rate pacing (400–450 bpm) for 4–8 weeks, with intensity calibrated to twice-threshold current (0.2 ms pulse width). AF success criterion: Irregular atrial rhythm ≥5 s.
⑤ Pigs follow similar chronic protocols (400 bpm ×6 weeks), often combined with transseptal/coronary sinus catheterization.
⑥ Sheep/Goats uniquely employ high-frequency intermittent bursts (50 Hz, 390 ms pulse width, 3–5 V) in 30 s on/off cycles for long-term maintenance (>6 weeks), occasionally supplemented by 900 bpm continuous pacing.
⑦ Horses use adaptive burst pacing (1–8 h/day) with automated rate support (≥170 bpm) to sustain AF.

#### 3.1.2 Transesophageal electrical stimulation

Transesophageal electrical stimulation has emerged as a significant method for electrophysiological induction in animal models of AF. This technique involves placing an electrode within the esophagus of an anesthetized animal, which allows high-frequency currents to penetrate the esophageal wall and stimulate left atrial cardiomyocytes, thereby triggering action potentials. Concurrently, electrical stimulation activates vagal nerve endings surrounding the esophagus, eliciting a vagal response. This response leads to the activation of M2 receptors on atrial cells through the release of acetylcholine, resulting in the enhancement of potassium channels and the inhibition of calcium channels. These alterations in ion channel function shorten the effective atrial refractory period and increase the dispersion of the refractory period, thereby facilitating the development of AF (58).

The electrophysiological techniques employed in transesophageal electrical stimulation models are largely similar to those used in direct atrial stimulation models. However, both approaches face the challenge of a lack of standardized electrophysiological stimulation parameters. In the context of indirect esophageal stimulation models for AF, most researchers opt for electrode placement within the esophagus. This method is less invasive and more aligned with animal welfare standards compared to the insertion of electrodes into the right atrium via the jugular vein. Despite its advantages, transesophageal electrical stimulation requires careful consideration of several parameters, including the frequency, duration, and intensity of the electrical stimulation, to ensure consistent and reliable induction of AF. The development of standardized protocols for this method would significantly enhance the reproducibility and comparability of research findings across different studies (Table 3).

**Table 3:** Af model induced by transesophageal electrical stimulation.

Author	Preprocessing	Electrophysiologic pacing parameters	Animal species	Definition of AF	Induction success rate	Scope of application
Zuo et al. (59)	-	Transesophageal burst rapid pacing, each mouse was stimulated 5 times continuously.	C57BL/6 Mice	≥1 s; Irregular	1/6; 16.7%	Gut microbiota.
Yang et al. (60)	-	Transesophageal burst pacing (specific parameters unknown).	C57BL/6 Mice	-	2/6; 33.3%	Mitochondrial function and calcium channels.
Zhan et al. (61)	Angiotensin II was subcutaneously injected at 2 mg/kg/d for 14 d.	Transesophageal burst pacing (specific parameters unknown).	C57BL/6 Mice	≥1 s; Irregular	9/10; 90%	Apoptosis and fibrosis.
Hai et al. (62)	Angiotensin II was injected by a subcutaneous osmotic pump at 2 mg/kg/d for 28 d.	Transesophageal burst pacing (specific parameters unknown).	C57BL/6 Mice	≥1 s; Irregular	-	Inflammation, oxidative stress, and fibrosis.
Jiang et al. (63)	-	Transesophageal burst pacing (specific parameters unknown).	C57BL/6 Mice	-	5/18; 27.8%	Calcium homeostasis.
Suffee et al. (64)	-	Transesophageal burst pacing was started at 10 Hz for 3 s.	C57BL/6J Mice	-	-	Metabolomic and lipidomic analysis.
Kondo et al. (65)	-	Transesophageal burst pacing (specific parameters unknown).	C57BL/6 Mice	≥1 s; Irregular	-	Inflammation, lipid metabolism, and fibrosis.
Liu et al. (66)	-	Electrical stimulation at a frequency of 450 stimuli per minute and a duration of 1 ms per stimulus. The pacing threshold was typically between 10 and 20 V. After establishing reproducible atrial pacing at twice the pacing threshold with a stimulation duration of 1 ms.	Wistar Rats	≥3 s; Irregular	2%–10%	Activation of sympathetic nervous system.
Mehdizadeh et al. (67)	-	Transesophageal burst pacing (specific parameters unknown).	Sprague-Dawley rats at 3 months	≥1 s; Irregular	1/10; 10%	Cellular senescence.
Mehdizadeh et al. (67)	-	Transesophageal burst pacing (specific parameters unknown).	Sprague-Dawley rats at 20 months	≥1 s; Irregular	10/10; 100%	Cellular senescence.

### 3.2 Chemical induction

Chemical induction represents one of the applicable methods for establishing AF models in small rodents such as mice and rats. The most common agent used are mixtures of acetylcholine (Ach) and calcium chloride (CaCl₂). Ach accelerates myocardial repolarization by activating potassium channels, thereby shortening the AERP, while CaCl₂ induces acute hypercalcemia, leading to dysregulation of calcium homeostasis in cardiomyocytes. The synergistic effects of these agents can trigger delayed afterdepolarizations and ectopic activity, as well as create heterogeneous cardiac conduction through partial blockade of conduction pathways, ultimately forming a functional reentry substrate. These electrophysiological alterations collectively establish an arrhythmogenic substrate that promotes AF initiation and maintenance (68).

Specifically, a mixture of Ach and CaCl_2_ is commonly administered intravenously via tail vein injection to rats or mice. The typical concentration ratios are 60 μg/ml Ach + 10 mg/ml CaCl_2_ or 66 μg/ml Ach + 10 mg/ml CaCl_2_. For rats, the usual dose is 0.1 ml per 100 g of body weight, and the administration period is typically 5–10 days, although some studies extend this to 14 or 28 days. However, care must be taken to avoid repeated intraperitoneal injections, as this may lead to peritonitis and potentially result in the death of the experimental animal.

The advantages of this method include its simplicity, high reproducibility, and the ability to modulate arrhythmia duration by adjusting the dosing protocol, making it a practical tool for investigating AF mechanisms. However, chemical induction of AF modeling may not fully replicate the complex electrophysiological and structural remodeling observed in human AF. Therefore, it can be used in conjunction with other modeling techniques to provide a comprehensive understanding of the disease (Table 4).

**Table 4:** Af model induced by chemistry.

Author	Preprocessing	Molding process	Animal species	Definition of AF	Induction success rate	Purpose of the study
Zou et al. (69)	Angiotensin II was injected by a subcutaneous osmotic pump at 750 ng/kg/min for 28 d.	Ach-CaCl_2_ mixture (25 μg/ml Ach + 6 mg/ml CaCl_2_) was injected through a tail vein for 30 min.	C57BL/6 Mice	≥1 s; Irregular	14/17; 82%	Late sodium current.
Shi et al. (70)	-	Ach-CaCl_2_ mixture (66 μg/ml Ach + 10 mg/ml CaCl_2_) was injected through a tail vein for 14 d.	Wild-Type Mice	-	-	Methylation.
Guo et al. (71)	-	Ach-CaCl_2_ mixture (10 mg/ml CaCl_2_) was injected through a tail vein once a day for 7 d.	Sprague-Dawley Rats	-	-	Pathway crosstalk.
Zhou et al. (72)	-	Ach-CaCl_2_ mixture (60 μg/ml Ach + 10 mg/ml CaCl_2_) was injected through a tail vein for 7 d.	Sprague-Dawley Rats	-	89%	Atrial remodeling and cardiac function.
Yue et al. (73)	-	Ach-CaCl_2_ mixture (66 μg/ml Ach + 10 mg/ml CaCl_2_) was injected through a tail vein once a day for 28 d.	Sprague-Dawley Rats	-	-	Cardiac fibrosis and inflammation.
Zhu et al. (74)	-	Ach-CaCl_2_ mixture (60 μg/ml Ach + 10 mg/ml CaCl_2_) was injected through a tail vein for 7 d.	Sprague-Dawley Rats	-; Irregular	-	Atrial remodeling and fibrosis.
Shi et al. (75)	-	Ach-CaCl_2_ mixture (66 μg/ml Ach + 10 mg/ml CaCl_2_) was injected through a tail vein once a day for 5 weeks.	Sprague-Dawley Rats	-	19/20; 95%	Calcium homeostasis.
Wang et al. (76)	-	Ach-CaCl_2_ mixture (66 μg/ml Ach + 10 mg/ml CaCl_2_) was injected through a tail vein once a day for 10 d.	Sprague-Dawley Rats	-	-	Paraventricular nucleus neurons activity.
Badreldin et al. (77)	-	Ach-CaCl_2_ mixture (60 μg/ml Ach + 10 mg/ml CaCl_2_) was injected through a tail vein for 7 d.	Wistar Rats	-	5/5; 100%	Oxidative stress, inflammation, apoptosis, and fibrosis.

### 3.3 Trauma induction

Trauma-induced AF models effectively replicate the pathogenesis of AF under various cardiac injury conditions, encompassing three principal approaches: aseptic pericarditis, mitral regurgitation, and chronic heart failure models (Table 5).

**Table 5:** Af model induced by trauma.

Author	Trauma	Molding process	Animal species	Definition of AF	Purpose of the study
Fu et al. (82)	Aseptic pericarditis	The surface of the atrium was dusted with talcum powder and a single layer of gauze was placed over it. Rapid stimulation (25, 30, 40, 50, and 83 Hz) was applied for 30 s in 5 consecutive bursts at 5 min intervals.	Sprague-Dawley Rats	≥1 s; Irregular	Inflammation and fibrosis.
Wu et al. (83)	Aseptic pericarditis	The surface of the atrium was dusted with talcum powder and a single layer of gauze was placed over it. Rapid stimulation (25, 30, 40, 50, and 83 Hz) was applied for 30 s in 5 consecutive bursts at 3 min intervals.	Sprague-Dawley Rats	≥5 s; Irregular	Inflammation and fibrosis.
Liao et al. (84)	Aseptic pericarditis	The surface of the atrium was dusted with talcum powder and a single layer of gauze was placed over it. Rapid stimulation (25, 30, 40, 50, and 83 Hz) was applied for 30 s in 5 consecutive bursts at 3 min intervals.	Sprague-Dawley Rats	≥1 s; Irregular	Inflammation, calcium channels, and fibrosis.
Zhang et al. (85)	Aseptic pericarditis	The surface of the atrium was dusted with talcum powder and a single layer of gauze was placed over it. Rapid atrial pacing was performed after surgery.	Goats	-	Inflammation.
McGilvray et al. (80)	Mitral regurgitation	Creation of mitral regurgitation by tendon cable avulsion.	Dogs	-	Experimental model.
Goldberg et al. (86)	Chronic heart failure	The ventricular pacemaker was activated in all dogs to induce HF by pacing the ventricles at 220 bpm for 28 d. After induction of HF, ventricular pacing was terminated and the atrial pacemaker was activated (600 bpm) and kept on to induce and maintain AF for the next 6 months.	Dogs	-	Vagal nerve stimulation.

#### 3.3.1 Aseptic pericarditis model

Aseptic pericarditis is a significant contributor to postoperative AF following cardiac surgery. It is hypothesized that a single reentrant loop with fibrillatory conduction may underlie this type of AF. This model is induced by surgically exposing the heart and applying inflammatory agents such as talcum powder, formaldehyde, or autologous blood on the pericardial surface, or by injecting these agents into the pericardial cavity via catheterization to provoke an inflammatory response (78). The aseptic pericarditis model is frequently used for trauma-induced AF due to its reliability in reproducing the inflammatory environment associated with postoperative AF.

#### 3.3.2 Mitral regurgitation model

Mitral regurgitation, a prevalent valvular disease, is closely associated with AF (79). Mitral regurgitation-induced AF models are generated via surgical valve injury, such as severing the chordae tendineae or perforating the leaflets, and catheter-based techniques like chordal clipping or implanting regurgitation-inducing devices. These approaches increase atrial volume overload and wall stress, promoting AF susceptibility (80). These models are invaluable for understanding how mitral regurgitation contributes to AF and for developing strategies to prevent or treat AF in patients with valvular heart disease.

#### 3.3.3 Chronic heart failure model

Chronic heart failure is a prevalent clinical condition that often precedes the development of AF. Chronic heart failure-induced AF models are developed using strategies that include inflicting myocardial injury, such as through coronary artery ligation, and administering cardiotoxic drugs, such as doxorubicin. These methods trigger atrial structural and functional remodeling, thereby establishing an electrophysiological environment that is highly susceptible to AF (81). This model is instrumental in studying the progression from chronic heart failure to AF and in developing therapeutic interventions aimed at preventing AF in patients with chronic heart failure.

### 3.4 Gene editing technology

Genetic editing technologies are predominantly employed to develop models that replicate the genetic underpinnings and pathological mechanisms of human AF. By employing precise gene-editing tools like CRISPR-Cas9, targeted genetic modifications are made in animal models or cellular systems to elicit electrophysiological irregularities, structural remodeling, or disruptions in calcium regulation. Gene-edited AF models are frequently examined using zebrafish and mice. These models facilitate the direct introduction of pathogenic mutations associated with AF, such as those in ion channel genes, calcium regulatory genes, and structural protein genes, thereby simulating human genetic mutations. Additionally, loss-of-function or gain-of-function models can be established through gene knockout or knock-in approaches. Moreover, the efficacy of gene therapy or chemical inductions can be evaluated by editing potential therapeutic targets (Table 6).

**Table 6:** Af models constructed by gene editing technology.

Author	Gene Editing	Type	Effect
Tucker et al. (87)	Morpholino-mediated knockdown of the PRRX1 orthologues in the zebrafish (prrx1a and prrx1b).	Zebrafish	PRRX1 suppression results in action potential shortening that may promote AF.
Jiang et al. (88)	Targeted ttna mutagenesis yields a 9-amino acid deletion within the Ig-107 structural domain of zebrafish titin using CRISPR-Cas9 technology.	Zebrafish	52% of ttna Δ9/Δ9 adult zebrafish demonstrate P-wave loss and irregular R-R intervals, indicating AF.
Ma et al. (22)	Zebrafish overexpressing the p.R355C mutant was established by microinjecting 25 pg of wTBX5 or mTBX5 RNA into zebrafish oocytes.	Zebrafish	22.2% of zebrafish with the overexpression of mutant TBX5 exhibited several rhythm disturbances including AF and conduction block.
Moreno-Manuel et al. (89)	Vectors encoding the SQTS3 Kir2.1 mutant (Kir2.1E299V) were packaged into adeno-associated virus (AAV) serotype 9 (AVV9) capsids. After anaesthesia, mice were administered 3.5 × 10^10^ viral genomes (vg) per animal i.v. in a final volume of 50 μl.	C57BL/6J mice	Kir2.1E299V mice are highly inducible for atrial arrhythmias.
Kim et al. (90)	Established paired-like homeodomain 2 transcription factor deficient (Pitx2+/−) mice.	Mice	Pitx2^+/−^ mice have increased susceptibility to AF.
Zhang et al. (91)	Sarcoplasmic reticulum Ca^2+^ leak model calsequestrin 2 R33Q (Casq2R33Q/R33Q) mice.	Mice	Casq2^R33Q/R33Q^ mice have increased susceptibility to AF.
Ozcan et al. (92)	Cardiac-specific liver kinase B1 (LKB1) knockout (KO) mice.	Mice	Spontaneous AF.
Ni et al. (93)	CREM-Ib*Δ*C-X transgenic (CREM) mice.	Mice	Spontaneous AF.
Xiao et al. (94)	The endogenous angiotensin-converting enzyme gene under the control of the alpha-myosin heavy chain promoter.	Mice	Spontaneous AF.
Saba et al. (95)	Heterozygous cardiomyocyte-specific transgenic mice overexpressing TNF-α under the αMHC promoter.	Mice	Spontaneous AF.

## 4 Construction of AF cellular models

Cellular models play an indispensable role in AF research, with primary cultured cardiomyocytes, HL-1, human-induced pluripotent stem cell-derived cardiomyocytes (hiPSC-CMs), and H9c2 cells being the most widely studied (Table 7).

**Table 7:** Cellular models of AF.

Author	Method	Molding process	Cell type
Liu et al. (110)	Electrical stimulation	The cardiomyocytes were stimulated at 6 Hz for 24 h, using the following parameters:1.5 V/cm field strength, square-wave, 5-ms pulses.	Primary cultured cardiomyocytes
Perike et al. (96)	Electrical stimulation	HL-1 cells were stimulated at 1 Hz for 3 s, using the following parameters: a voltage of 15 V.	HL-1 cells
Yuan et al. (111)	Electrical stimulation	HL-1 cells were stimulated at 5 Hz, using the following parameters: 1.5 V/cm field strength, square-wave, 10-juti er ms pulses.	HL-1 cells
Li et al. (40)	Electrical stimulation	HL-1 cells were stimulated at 10 Hz, using the following parameters: 1.5 V/cm field strength, square-wave, 5-ms pulses.	HL-1 cells
Adili et al. (10)	Electrical stimulation	HL-1 cells were stimulated at 5 Hz for 12 or 24 h, using the following parameters: a voltage of 3 V, 5-ms pulses.	HL-1 cells
Seibertz et al. (112)	Electrical stimulation	iPSC-aCMs were stimulated at 1 or 3 Hz for 24 h, using the following parameters: 5-ms pulses.	iPSC-aCMs
Hutschali et al. (113)	Cell co-culture	Co-cultures of iPSC-aCMs and M1 macrophages.	iPSC-aCMs
Brown et al. (114)	Cell co-culture	Co-cultures of iPSC-aCMs and atrial fibroblasts	iPSC-aCMs
Schulz et al. (115)	Gene editing	CRISPR/Cas9 was used to delete PITX2 in a healthy human iPSC line.	iPSC-aCMs
Hong et al. (116)	Gene editing	Edited iPSCs heterozygous for SCN5A p.E428K using CRISPR-Cas9.	iPSC-aCMs
Liu et al. (117)	Electrical stimulation	The cells were stimulated with an electric field of 1.0 v/cm and a frequency of 10 Hz for 12–72 h.	H9c2 cells
Lu et al. (118)	Electrical stimulation	Cells receive continuous electrical stimulation of 0.2 V/cm for 24 h.	H9c2 cells
Lu et al. (118)	Gene editing	Mitochondrial calcium uniporter (MCU) knockout using CRISPR-Cas9.	H9c2 cells
Luo et al. (119)	Gene editing	Transfect silencing miR-26.	H9c2 cells

### 4.1 HL-1 cells

HL-1 cells, a lineage derived from AT-1 mouse atrial tumor cardiomyocytes, retain intrinsic contractility and key electrophysiological properties of cardiomyocytes, making them ideal for investigating AF-related electrophysiological dysfunction, molecular mechanisms, and drug screening. Rapid electric field stimulation of HL-1 cells can induce a rapid pacing state, effectively recapitulating early AF-associated remodeling phenomena (96). Due to their reliability and reproducibility, HL-1 cells have gained the status of the most widely utilized cellular model in AF research, providing a robust platform for pathophysiological studies and therapeutic development.

HL-1 cells, while valuable for atrial fibrillation (AF) modeling, have inherent limitations. Firstly, their origin from a tumor raises concerns. The HL-1 cell line was derived from an AT-1 subcutaneous tumor in an adult female C57BL/6J mouse, which was part of a transgenic model expressing the SV40 large T antigen. This antigen induces transformation and immortalization of cardiomyocytes, endowing the cells with tumor-associated characteristics (97). Secondly, the cells exhibit limitations in structural maturity. Originating from the atrium, HL-1 cells reflect the ultrastructure of embryonic atrial myocytes, featuring a disorganized arrangement of myofibrils that does not replicate the structured organization of mature cardiomyocytes, particularly those of the ventricle (98). Lastly, there are significant electrophysiological discrepancies between species. The expression of crucial ion channels in mouse-derived HL-1 cells, such as the L-type calcium channels, diverges markedly from that in humans (99). This discrepancy limits the cells' applicability in directly studying the pathophysiological mechanisms of human AF.

### 4.2 Human induced pluripotent stem cell-derived cardiomyocytes (hiPSC-CMs)

HiPSC-CMs provide an unlimited human cell source for modeling atrial fibrillation *in vitro*, enabling the generation of 3D cardiac organoids that recapitulate structural and functional tissue complexity. When integrated with electrical stimulation, these systems synchronously simulate electrical remodeling and structural alterations, offering a powerful platform for cardiac electrophysiological investigations (100, 101). However, hiPSC-CMs exhibit fetal-like electrophysiological properties-including shortened action potential duration and reduced contractility-limiting their direct applicability to adult disease pathophysiology (102, 103). Despite these limitations, maturation strategies-such as chronic electromechanical stimulation, biochemical induction (thyroid hormone/fatty acids), and 3D co-culture with fibroblasts-significantly enhance functional maturity, bridging the translational gap for studying adult-onset arrhythmogenesis (100, 103). In addition, technical challenges (e.g., complex differentiation protocols requiring specialized equipment like C-PACE pacing systems) and high costs further restrict broad implementation.

Although the clinical application of hiPSC-CMs is still in its early stages, their potential for cardiac regeneration and repair has garnered significant attention. A research team successfully modified the electrical conduction and overall heart function in a porcine myocardial infarction model by co-culturing hiPSC-CMs with vascular cells to create vascularized engineered cardiac microtissues and then transplanting them (104). Additionally, another study developed high cell density engineered cardiac tissue through the large-scale expansion of hiPSC-CMs, which was successfully transplanted into a porcine heart with chronic ischemia, with the successful delivery of 1 billion hiPSC-CMs (105). These pivotal findings not only substantiate the therapeutic potential of hiPSC-CMs but also establish a robust foundation for the clinical translation of engineered cardiac tissues.

### 4.3 H9c2 cells

H9c2 cells, derived from a rat embryonic ventricular myoblast cell line, have been used in the construction of *in vitro* AF models due to their preservation of some cardiomyocyte characteristics, including contractile function, morphological features, and metabolic pathways (106). Pathological processes associated with AF, such as electrical remodeling and calcium homeostasis imbalance, can be partially mimicked in H9c2 cells through electrical stimulation or chemical induction (e.g., adriamycin, hyperglycemic/hypoxic stress). This makes H9c2 cardiomyocytes an ideal model for studying cardiac diseases, drug screening, and cardiac physiological mechanisms.

The easy immortalization properties of H9c2 cells support low-cost, large-scale culture and high gene-editing efficiency, making them suitable for rapid target validation. H9c2 cells also retain a variety of characteristic developmental markers of cardiomyocytes, such as the expression of cardiac-specific proteins troponin T and myosin heavy chains, making them invaluable in studies of cardiac development, functional regulation, and disease mechanisms (106, 107). Recognized for their robust responses to drugs and stressors, H9c2 cells are widely used in drug screening and toxicological evaluations (108).

However, H9c2 cells also have significant drawbacks. Although they share some common features with primary cardiomyocytes, H9c2 cells cannot fully replicate the complexity of adult cardiac tissues in terms of genetic and functional properties. They are significantly different from both primary neonatal cardiomyocytes and adult cardiomyocytes (109). Additionally, this cell line exhibits marked genetic instability, with chromosome numbers fluctuating between 74 and 81, often presenting a triploid state. Chromosome deletions and extra copies are common, and this genetic instability may impact the reproducibility of experimental results (106). Moreover, the structural features of H9c2 cells are limited by their embryonic origin. They lack the highly ordered structure of mature cardiomyocytes and do not express atrial-specific markers (e.g., Nppa, Nppb, Nppc) (106). These limitations further affect the applicability of the model, particularly in studies requiring precise simulation of human AF electrophysiology.

## 5 Evaluation of AF models

Establishing rigorous and comprehensive evaluation criteria is essential for studying AF models to ensure the reliability and consistency of research findings. This study highlights the importance of assessing electrophysiological properties, atrial functional metrics, and biomarkers in evaluating AF models. These parameters are crucial for understanding the pathophysiology of AF and developing effective therapeutic interventions.

### 5.1 Evaluation of electrophysiological properties

Electrophysiological assessment is crucial for evaluating the success of AF models. The electrocardiogram (ECG) serves as the primary diagnostic tool, with typical ECG manifestations of AF in animal models including (1) the absence of the P wave, replaced by rapid, irregular fibrillatory (f) waves, and (2) absolute inequality of RR intervals (120). Key electrophysiological parameters for model evaluation include the AF triggering rate, the duration of individual AF episodes, the effective duration of AF episodes, the conduction velocity of electrical impulses in atrial muscle, and the electrophysiological heterogeneity across different atrial regions. These parameters are the most direct indicators of model success (121).

A high evoked rate suggests that the model effectively simulates the susceptibility to AF. The duration of AF episodes reflects the characteristics of AF maintenance. A shortened atrial effective refractory period is a hallmark of atrial electrical remodeling and is closely associated with increased susceptibility to AF initiation and maintenance, while slowed conduction velocity and increased conduction heterogeneity are key factors promoting AF persistence during episodes (28). These electrophysiological properties are critical for assessing the reliability and validity of AF models, ensuring they accurately represent the pathophysiological mechanisms of the condition.

### 5.2 Evaluation of atrial structure and function

Echocardiography is a pivotal tool for assessing changes in cardiac structure and function within AF models. A significant pathological feature observed in these models is the enlargement of both the left and right atria, which is a hallmark of AF. This method allows for the evaluation of atrial systolic function, ventricular systolic and diastolic function, and hemodynamic alterations, providing insights into the impact of AF on overall cardiac performance.

### 5.3 Evaluation of biomarkers

#### 5.3.1 Indicators of inflammation

Inflammatory responses are integral to the progression of cardiovascular diseases and significantly elevate the risk of developing AF (122). Systemic inflammation, characterized by the release of inflammatory cytokines and activation of the immune system, induces underlying pathologies such as hypertension, atherosclerosis, and vascular remodeling. Moreover, it directly impacts myocardial tissues, promoting AF by causing cellular necrosis and altering cardiomyocyte properties (123, 124). In this context, macrophages are instrumental as key effector cells of the inflammatory response. Upon exposure to injury, infection, or other pathological stimuli, M1 macrophages are activated, releasing substantial amounts of pro-inflammatory factors like TNF-α. They also activate signaling pathways such as NF-κB and MAPK, which in turn promote the release of inflammatory mediators including IL-6 and IL-1β (125). TNF-α not only further activates the NF-κB pathway by binding to its receptors TNFR1/2, forming a positive feedback loop that continuously enhances the transcriptional expression of its own and other inflammatory factors like IL-6, but also significantly boosts the activity of the NLRP3 inflammasomes. This amplifies the release of IL-1β, contributing to fibrosis (126). Consequently, IL-1β and IL-6 serve as pertinent biomarkers for AF progression (127, 128).

#### 5.3.2 Indicators of myocardial fibrosis

The pathogenesis of myocardial fibrosis in AF involves the complex interplay of multiple signaling pathways, with the TGF-β1/Smad signaling pathway being a central regulatory hub (129). Research indicates that angiotensin II serves as a potent activator of the TGF-β1/Smad pathway (62). The TGF-β1 receptor TβR II activates TβR I kinase, leading to the phosphorylation of Smad2/3. These phosphorylated Smads then complex with Smad4 and translocate to the nucleus, where they regulate the transcription of target genes such as Collagen I and Collagen III (130, 131). The accumulation of these collagen proteins contributes to ventricular remodeling and myocardial fibrosis. Consequently, TGF-β1, p-Smad2, p-Smad3, collagen I, and collagen III are recognized as crucial biomarkers of atrial fibrosis.

Matrix metalloproteinases, a family of enzymes capable of degrading nearly all extracellular matrix proteins, play a significant role in the structural remodeling of both normal and pathological tissues. Tissue inhibitors of metalloproteinases are endogenous specific inhibitors of matrix metalloproteinases and can modulate tissue remodeling by inhibiting MMP activity (132). An imbalance between matrix metalloproteinases and tissue inhibitors of metalloproteinases can lead to excessive fibrotic processes (133). Gelatinases, specifically MMP-2 and MMP-9, have the unique ability to degrade denatured collagen types I, II, and III and gelatin, playing a pivotal role in collagen remodeling within the extracellular matrix (134, 135). TIMP-2 specifically inhibits MMP-2, while TIMP-1 inhibits MMP-9 (136, 137). Therefore, MMP-2, MMP-9, TIMP-1, and TIMP-2 have been established as important biomarkers for assessing the degree of atrial fibrosis due to their key regulatory roles in extracellular matrix remodeling.

#### 5.3.3 Indicators of ion channels and currents

The initiation and perpetuation of AF are intricately tied to the abnormal function of ion channels within atrial myocytes, leading to dysregulation of key ionic currents. Central to the pathophysiology of AF is electrical remodeling, a process wherein modifications in ion channel expression or function result in substantial alterations to action potential characteristics and the underlying ionic currents (138). The fast sodium current (INa), crucial for regulating atrial conduction velocity as the principal current responsible for the rapid depolarization phase (phase 0) of the action potential, is diminished during AF due to abnormalities in the sodium channels (e.g., Nav1.5). These sodium channel anomalies include accelerated inactivation and delayed recovery, leading to a significant reduction in INa density. This reduction in INa in turn slows conduction velocity and can contribute to enhanced refractoriness, facilitating the onset of AF (139).

A hallmark of electrical remodeling is the shortening of action potential duration, a phenomenon primarily driven by changes in the magnitude and kinetics of key repolarizing currents, resulting from an altered balance between the inward L-type calcium current (ICa,L) and various outward potassium currents (140, 141). Downregulation of L-type calcium channels reduces ICa,L, while upregulation of specific potassium channels increases currents such as the inwardly rectifying potassium current (IK1, conducted by Kir2.x channels) and the rapidly activating delayed rectifier potassium current (IKr, conducted by hERG/KCNH2 channels). Both the decrease in ICa,L and the increase in IK1 and IKr can result in action potential duration shortening (142, 143). In certain experimental models, the slow-activating delayed rectifier potassium current (IKs, conducted by KCNQ1/KCNE1 channels) also participates in action potential repolarization. Dysfunction of channels carrying IKr or IKs can precipitate early afterdepolarizations, serving as a significant trigger for AF (32).

In summary, the functional status of critical ion channels (e.g., Nav1.5, Cav1.2, Kir2.x, hERG, KCNQ1) and the corresponding ionic currents they carry (INa, ICa,L, IK1, IKr, IKs) serve as vital indicators for evaluating electrical remodeling in AF. Monitoring the expression, function, and regulation of these channels and the currents they generate provides essential insights into the electrical underpinnings of the condition and can guide therapeutic interventions aimed at normalizing cardiac electrical activity.

#### 5.3.4 Neurohormone levels

The progression of AF is intricately linked to the abnormal activation of multiple neurohormonal systems, which collectively promote atrial electrical and structural remodeling through complex interactions. The renin-angiotensin-aldosterone system is among the most critical regulatory pathways. Its core effector molecule, Angiotensin II, fosters myocardial fibrosis by activating the TGF-β1/Smads signaling pathway (138). Concurrently, Angiotensin II enhances L-type calcium channel activity, leading to intracellular Ca^2+^ overload. This overload shortens the plateau phase of atrial action potentials and accelerates atrial electrical remodeling (144, 145). Aldosterone, the end effector hormone of renin-angiotensin-aldosterone system, contributes to cardiac oxidative damage by binding to the mineralocorticoid receptor in cardiomyocytes and fibroblasts. It upregulates the expression of angiotensin II type 1 receptor (AT1R) and angiotensin-converting enzyme, amplifying the Angiotensin II signaling pathway. Additionally, aldosterone regulates the transcription of pro-atherogenic and oxidative stress-related genes, inflammatory responses, and fibrotic processes, providing a significant pathological substrate for AF (146, 147).

The sympathetic nervous system also plays a crucial role in AF development. Epinephrine significantly alters atrial electrophysiological properties by activating β1 and α1 adrenergic receptors. This activation involves the G-protein-coupled inwardly rectifying potassium channels and the formation of calcium overload. Norepinephrine increases intracellular sodium load by enhancing Na^+^/Ca^2+^ exchanger activity, induces delayed afterdepolarization, and promotes the formation of localized ectopic foci of agitation, collectively constituting a triggering mechanism for AF development (58).

Furthermore, endothelin-1, a key mediator of the inflammatory response, significantly increases collagen deposition and interstitial fibrosis by promoting the secretion of platelet-derived growth factor-B (PDGF-B) by atrial fibroblasts (148). Clinical studies have also found that brain natriuretic peptide (BNP) levels are strongly associated with AF prognosis (149). Their elevation not only reflects atrial pressure and volume overload but may also promote AF recurrence after radiofrequency ablation by affecting ventricular function. These neurohormonal cascade responses form a complex regulatory network that collectively promotes the development and maintenance of AF through multiple pathways, including electrical remodeling, structural remodeling, and autonomic regulation (150).

## 6 Bioengineering

### 6.1 Three-dimensional (3D) engineered tissues

3D engineered tissues are able to more closely resemble the structure and function of human tissues by assembling induced pluripotent stem cell (hiPSC)-derived cardiomyocytes into three-dimensional structures. This model provides a platform to study the pathophysiological mechanisms of AF in an environment that more closely mimics physiological reality. 3D engineered tissues are capable of mimicking the electrophysiological properties of cardiomyocytes, intercellular interactions, and mechanical properties of tissues, thus more accurately reflecting the physiological and pathological states of cardiac tissues (151, 152). Currently, 3D engineered tissues are used to simulate the electrophysiological properties of cardiac tissues, particularly in the fields of atrial-like organs, patient-specific models, and 3D hydrogels.

#### 6.1.1 Atrial-like organs

Atrial-like organs are an emerging *in vitro* model that mimics the structure and function of atrial tissue by assembling hiPSC-derived cardiomyocytes into 3D structures. These organs are capable of generating spontaneous and induced electrical activity with higher conduction velocities than 2D cultures and can be used to study complex electrophysiological disorders, such as refractory arrhythmias and inherited arrhythmias (151). This provides new perspectives for understanding the pathomechanisms of AF, drug screening, and personalized therapy.

#### 6.1.2 Patient-specific models

3D models constructed based on patient MRI or CT data accurately reflect the geometry and fibrosis of the atria. These models are used to support ablation therapy for patients with atrial fibrillation, improving patient prognosis by increasing the safety and success of the procedure (153). MRI-based 3D reconstruction technology can accurately differentiate fibrotic tissue from normal myocardial tissue by acquiring cross-sectional images of the heart combined with advanced image segmentation algorithms to generate detailed 3D anatomical structural models. This technique provides an important structural basis for the study of electrophysiological mechanisms of atrial fibrillation (154).

#### 6.1.3 3D hydrogels

Hydrogels are cross-linked, water-soluble polymers that allow cells to be embedded into the three-dimensional structure of the gel. 3D hydrogels are a hydrogel-based 3D engineered cardiac tissue model that mimics the structure and function of cardiac tissue (155). This model supports the contractile function of cardiomyocytes, exhibiting an ordered myofibrillar structure and a well-defined beating pattern, and can be used as a test bed for drug screening and ablative therapies (156).

Despite their ability to mimic many cardiac tissue properties, hiPSC-derived cardiomyocytes typically exhibit fetal-like structural features, electrophysiological properties, and metabolic properties, which limits their use in mimicking adult atrial fibrillation (101, 102). Additionally, the intercellular interactions in 3D engineered tissues, although complex, still do not fully mimic the intercellular signaling and microenvironment found *in vivo*. The construction and use of 3D engineered tissues are technically demanding and require precise cell culture and tissue engineering technology support.

### 6.2 Artificial intelligence and computational simulation

In recent years, Artificial Intelligence (AI), particularly Machine Learning and Deep Learning, has rapidly emerged as a transformative tool in AF care. AI has demonstrated significant applications in early detection, risk prediction, treatment optimization, and remote monitoring of AF (157).

In clinical diagnostics, AI can significantly improve the early detection of AF by analyzing ECG and wearable device data. By analyzing RR interval differences and the presence or absence of P waves in ECGs, AI can identify AF with up to 99.2% accuracy (158). Additionally, AI models based on photovolumetric pulse wave signals (PPG) combined with wearable devices enable real-time dynamic monitoring of AF (159). This technique not only improves the detection rate of AF but also creates favorable conditions for early diagnostic intervention in asymptomatic patients.

On the therapeutic side, AI provides assistance in personalized anticoagulation decisions, catheter ablation planning, and optimizing antiarrhythmic drug selection (157). By analyzing cardiac imaging data, AI can predict non-pulmonary vein triggers for atrial fibrillation, thereby improving the success rate of ablation therapy (160). Moreover, AI can assist in optimizing drug selection by predicting a patient's response to or risk of side effects from specific antiarrhythmic drugs (161).

Machine learning models have also made significant progress in predicting the risk of developing atrial fibrillation after cardiac surgery. These models are an important tool for predicting the occurrence of AF after cardiac surgery (162).

## 7 Discussion

Currently, AF animal models are primarily categorized into small animals, large animals, and model organisms, with small animal models, particularly mice and rats, being the most extensively used in basic research. In terms of experimental design, electrophysiological induction is the most common method for inducing AF in small animal models. However, due to the small size of mouse hearts and the short conduction pathways, it is challenging to form stable reentrant circuits, leading to a generally low AF induction rate using electrophysiological induction alone. Consequently, most researchers opt to precondition the mice with Angiotensin II to enhance the model's success rate, which has been shown to significantly increase. This may be related to Angiotensin II triggering the activation of the NLRP3 inflammasome through a Ca^2+^-dependent mechanism, resulting in the secretion of IL-1β, thereby increasing oxidative stress levels in atrial cells, inducing cardiomyocyte hypertrophy and apoptosis, and consequently enhancing AF susceptibility (163).

In AF models of rats, the chemical induction method is often the preferred approach due to its simplicity, low cost, and good reproducibility. However, the chemical induction method showed a relatively low efficiency of AF induction. The main reasons for this phenomenon are as follows: This chemical induction method primarily works through acute electrophysiological perturbations-Ach activates muscarinic receptors to briefly shorten the atrial effective refractory period, while CaCl_2_ triggers premature electrical activity by increasing calcium ion influx. However, this mechanism of action has significant limitations: firstly, the duration of the drug's effect is short-lived; secondly, it cannot cause sustained myocardial damage; more importantly, the duration of atrial fibrillation induced by the Ach-CaCl_2_ protocol shows significant individual variability and fails to simulate the progressive structural remodeling characteristic of clinical chronic atrial fibrillation. Sprague-Dawley rats are relatively small in size, facilitating manipulation, and are comparatively less expensive, making them suitable for large-scale experimental research.

For establishing models in large animals, electrophysiological inductions are typically conducted using epicardial electrodes or transesophageal electrodes, eliminating the need for open-chest surgery or long-term drug administration. This reduces the impact of surgical complications and drug side effects on the animals, helping to maintain the stability and health of the animal models, and thus more accurately observe the pathophysiological changes associated with AF. Although some studies have attempted to induce AF by directly stimulating the vagus nerve trunk with high-frequency electrical stimulation, research indicates that low-intensity vagal nerve stimulation may actually play a protective role in the atria by regulating autonomic balance (164, 165). This biphasic effect, which is dependent on the intensity of stimulation, may be the primary reason limiting the application of vagus nerve trunk stimulation methods in AF research, and thus it is not recommended as a modeling method in this study.

Among existing traumatic AF models, the aseptic pericarditis model stands out due to its excellent reproducibility and clear inflammation-mediated mechanisms, making it a primary method for studying the relationship between atrial inflammation and the occurrence of AF. However, while the inflammatory response in aseptic pericarditis induces certain electrophysiological changes in the atria, additional electrophysiological inductions are still required to consistently trigger AF (85). Therefore, most researchers combine disease simulation with electrophysiological inductions to induce AF. Similarly, in mitral regurgitation-induced AF models, although chordal rupture surgery can successfully establish valvular AF models, the pathological process is relatively slow, often taking weeks to months to observe a stable AF phenotype. To accelerate model establishment and improve experimental efficiency, electrophysiological inductions can be combined postoperatively to actively induce electrical remodeling, significantly shortening the time to AF onset (80). Traumatic AF models usually require model establishment through surgical procedures or mechanical heart injury, which can cause significant damage to the animal, are more complex to perform, and may lead to severe inflammatory responses and secondary pathological changes. Thus, their application in the construction of pure AF models is somewhat limited and is more suitable for research on cardiovascular diseases complicated with other conditions.

In the context of AF cellular models, primary cultured cardiomyocytes, HL-1 cells, hiPSC-CMs, and H9c2 cells are most frequently utilized. HL-1 cells are particularly suited for high-throughput electrophysiological screening due to their ability to mimic early AF remodeling through rapid electrical pacing; however, their tumor origin, embryonic-like ultrastructure, and murine-specific ion channel expression significantly limit their translational relevance to human AF. HiPSC-CMs offer unparalleled human genetic fidelity, enabling patient-specific disease modeling, and 3D organoids derived from hiPSC-CMs can recapitulate tissue-level complexity, providing a more accurate representation of human cardiac tissue; yet, their fetal-like electrophysiological properties necessitate maturation protocols to better simulate adult cardiac tissue, and the high cost and technical complexity associated with hiPSC-CMs can impede scalability. H9c2 cells, on the other hand, provide a cost-effective immortalized lineage that supports rapid gene editing and large-scale drug toxicity screening; however, their embryonic ventricular origin lacks atrial-specific markers, and genetic instability can compromise reproducibility.

In summary, the establishment of AF models is of paramount importance for delving into the pathogenesis of AF and exploring therapeutic strategies. Currently, research into treatment plans for human AF primarily relies on animal models and cellular models. Given the variability among different models and modeling methodologies, constructing appropriate AF models, optimizing their application, and investigating treatment plans that are more applicable to human AF require further research and systematic elucidation.

Small animals offer low cost, fast reproduction, and convenient gene editing, but their small heart size and electrophysiological differences limit their ability to form stable reentrant circuits and simulate chronic remodeling. Large animals, with hearts similar to humans, can spontaneously develop persistent AF, making them ideal for translational research, though they are costly and require complex surgeries. The trauma model in large animals clearly simulates AF causes like inflammation but can trigger systemic issues that complicate pure AF studies. Future directions include developing gene-edited large animals (e.g., porcine models) with human-specific mutations, applying organ microarrays and multi-omics analyses for greater accuracy, and establishing a unified phenotypic evaluation system for AF.

The optimal modeling of future AF requires hierarchical integration of cellular models, utilizing HL-1 and H9c2 cells for preliminary target validation and toxicity screening, while employing hiPSC-CMs for in-depth human-pathophysiological investigations and personalized therapeutic discovery. Cross-validation of findings across these models is essential to mitigate biases related to species-specific or maturity-related differences. Future advances in organ-on-chip technologies and machine learning-driven phenotype analysis are expected to further bridge the gap between cellular models and the clinical complexity of AF, enhancing the accuracy and efficiency of AF research and treatment strategies.
